# Lingonberry (*Vaccinium vitis-idaea* L.) Exhibits Antidiabetic Activities in a Mouse Model of Diet-Induced Obesity

**DOI:** 10.1155/2014/645812

**Published:** 2014-06-10

**Authors:** Hoda M. Eid, Meriem Ouchfoun, Antoine Brault, Diane Vallerand, Lina Musallam, John T. Arnason, Pierre S. Haddad

**Affiliations:** ^1^Natural Health Products and Metabolic Diseases Laboratory, Department of Pharmacology, University of Montreal, Station Centre-Ville, P.O. Box 6128, Montreal, QC, Canada H3C 3J7; ^2^Phytochemistry, Medicinal Plant and Ethnopharmacology Laboratory, Department of Biology, University of Ottawa, Ottawa, ON, Canada K1N 6N5; ^3^Canadian Institutes of Health Research Team in Aboriginal Antidiabetic Medicines and Montreal Diabetes Research Center, Canada; ^4^Department of Pharmacognosy, University of Beni Suef, Beni Suef 62511, Egypt

## Abstract

*Vaccinium vitis-idaea*, commonly known as lingonberry, has been identified among species used by the Cree of Eeyou Istchee of northern Quebec to treat symptoms of diabetes. In a previous study, the ethanol extract of berries of *V. vitis-idaea* enhanced glucose uptake in C2C12 muscle cells via stimulation of AMP-activated protein kinase (AMPK) pathway. The purpose of this study was to examine the effect of plant extract in a dietary mouse model of mild type 2 diabetes. C57BL/6 mice fed a high-fat diet (HFD, ∼35% lipids) for 8 weeks that become obese and insulin-resistant (diet-induced obesity, DIO) were used. Treatment began by adding *V. vitis-idaea* extract to HFD at 3 different concentrations (125, 250, and 500 mg/Kg) for a subsequent period of 8 weeks (total HFD, 16 weeks). The plant extract significantly decreased glycemia and strongly tended to decrease insulin levels in this model. This was correlated with a significant increase in GLUT4 content and activation of the AMPK and Akt pathways in skeletal muscle. *V. vitis-idaea* treatment also improved hepatic steatosis by decreasing hepatic triglyceride levels and significantly activated liver AMPK and Akt pathways. The results of the present study confirm that *V. vitis-idaea* represents a culturally relevant treatment option for Cree diabetics and pave the way to clinical studies.

## 1. Introduction

Obesity is a complex and multifaceted disorder. Given its current global epidemic stature and strong link to life-threatening illnesses such as diabetes, cardiovascular diseases, and cancers, the need to prevent or treat obesity and its complications has become more urgent.

Insulin resistance usually precedes the development of type 2 diabetes and is more common in obese individuals. In skeletal muscle, insulin promotes glucose uptake by activating the phosphatidylinositol-3 kinase (PI3-K)/Akt pathway and by inducing the translocation of glucose transporter GLUT4 from intracellular storage vesicles to the plasma membrane [1]. An alternative pathway for stimulating glucose uptake is the AMP-activated protein kinase (AMPK) pathway. AMPK stimulates GLUT4 translocation to the plasma membrane by a mechanism distinct from the PI3-K pathway stimulated by insulin [2]. It is of note that AMPK upregulates the expression of GLUT4, possibly through the direct phosphorylation of the transcriptional coactivator PPAR*γ* coactivator-1*α* (PGC-1*α*). On the other hand, activation of AMPK decreases intramyocyte accumulation of lipids and increases insulin sensitivity of muscle through phosphorylation and inhibition of acetyl-CoA carboxylase (ACC) [3, 4].

In the liver, AMPK decreases hepatic glucose production mainly by inhibiting the expression of gluconeogenic genes such as phosphoenolpyruvate carboxylase (PEPCK) and glucose 6-phosphate (G-6-Pase). Moreover, activation of AMPK stimulates fatty acid oxidation and inhibits expression of genes encoding lipogenic enzymes (fatty acid synthase and ACC) [5].

SIRT1 is another critical player in mammalian energy homeostasis. It is a nicotinamide adenine dinucleotide- (NAD+-) dependent deacetylase and a member of the mammalian sirtuin family. SIRT1 controls a variety of cellular processes such as apoptosis, cell cycle, and metabolism through deacetylation of target proteins including p53, NFkB, and PGC-1*α*. It is activated by fasting and caloric restriction as well as by many small molecules such as the plant phenols quercetin, piceatannol, and resveratrol. Activation of SIRT1 was reported to improve glucose homeostasis, increase insulin sensitivity, and improve mitochondrial function in skeletal muscle of rodent models of type 2 diabetes [6]. Conversely, activation of SIRT1 in the liver increases gluconeogenic genes and represses glycolysis suggesting that SIRT1 induces organ-specific metabolic responses. Similar to skeletal muscle, SIRT activation in liver promotes fatty acid oxidation and prevents diet-induced hepatic steatosis and insulin resistance [7].

Finally, peroxisome proliferator activated receptor-*α* (PPAR-*α*) belongs to the superfamily of PPAR nuclear receptors and is highly expressed in tissues with active fat metabolism such as liver, heart, and skeletal muscle. PPAR-*α* induces the expression of genes controlling the *β*-oxidation of fatty acids.

Type 2 diabetes has reached unprecedented proportions across Aboriginal populations all over the world. In Canada, the rates have risen exponentially above the national average over the past few decades and are expected to keep rising. For instance, the prevalence of diabetes among the Cree Nations of Eeyou Istchee (CEI) inhabiting the Eastern James Bay region of northern Quebec has tripled for adults aged over 20 years in the same time period [8]. To address this serious health issue facing Canadian First Nations, specifically the CEI, our research team aimed to identify culturally relevant treatments for diabetes within their traditional pharmacopeia.

Lingonberry (*V. vitis-idaea*) belongs to the Ericaceae plant family and is closely related to highbush blueberries (*Vaccinium corymbosum *L.) and cranberries (*V. macrocarpum *L.) [9]. The berries are edible and are used in northern Europe to make jams, sauces, and other foods [10]. They are also used traditionally as food by Indigenous Peoples of Canada where they are eaten raw, stewed, and served with fish or meat or mixed with boiled fish eggs, liver, and fat [11, 12]. The Cree use the berries as a folk medicine to treat frequent urination and other symptoms of diabetes [13, 14].

In a previous study, we reported that the ethanolic extract of the berries of* V. vitis-idaea* revealed interesting glucose uptake enhancing properties in cultured C2C12 skeletal muscle cells through the activation of AMPK [15]. In the present work, we assessed the effect of* V. vitis-idaea* extract in a mouse model of diet-induced obesity (DIO), which closely mimics human metabolic syndrome and early type 2 diabetes linked to unhealthy lifestyle. The most studied experimental model of DIO is the C57Bl/6J mouse strain. This strain becomes obese, insulin-resistant, and hyperglycemic when fed a high-fat diet [16]. Over and above systemic parameters of glucose and lipid homeostasis, we also paid attention to the major tissue components of insulin-dependent and -independent pathways described previously.

## 2. Materials and Methods

### 2.1. Plant Materials

Berries of* V. vitis-idaea* were harvested in the Eastern James Bay region, QC, Canada, according to traditional procedures (season, time of day, location, and gift offering) instructed by Cree elders. They were kept in dry cold place until used. Botanical identity was confirmed by Dr. Alain Cuerrier (Institut de Recherche en Biologie Végétale, Université de Montréal) and voucher specimens were deposited at the Montreal Botanical Garden Herbarium (voucher number Whap04-21). The 80% ethanolic extract was prepared as previously described [17] following standard operating procedures of Professor Arnason's laboratory.

### 2.2. Animals and* In Vivo* Experimental Protocols

Four-week-old male C57BL/6 mice were purchased from Charles River (St-Constant, QC). After acclimation, mice were randomly divided into five groups (*n* = 12 each) and started on regular chow (CHOW control group) or high-fat diet (35% fat, 20% protein, and 36.5% carbohydrate, Bio-Serv, Frenchtown, NJ, USA). After 8 weeks on these diets, the HFD-fed mice were obese and insulin-resistant. They weighed an average of 31.78 g ± 2.71 while their CHOW-fed counterparts weighed 24.8 ± 2.07 g. At this point, one group of HFD-fed mice served as DIO control (continued HFD intake for another 8 weeks), whereas the other three groups of HFD-fed mice received* V. vitis-idaea* extract at 3 doses (125, 250, and 500 mg/kg) incorporated in HFD for another period of 8 weeks.

Body weight, food intake, water intake, and blood glucose level were measured from nonfasting animals 2 or 3 times per week throughout the study. Tail blood was collected for glucose determination using a glucometer (Accu-Check Roche, Montreal, QC). At the end of the treatment study, animals were sacrificed and various tissues were harvested, weighed, and processed for subsequent analysis. All procedures and experimental protocols were authorized by the Université de Montréal Animal Experimentation Ethics Committee and respected guidelines of the Canadian Council for the Care and Protection of Animals.

### 2.3. Measurement of Plasma Samples

Plasma triglyceride, total cholesterol, LDL, HDL, alanine aminotransferase (ALT), aspartate aminotransferase (AST), alkaline phosphatase, and creatinine were assessed using standard clinical biochemistry protocols at Sainte-Justine's Children's Hospital (Montreal, Quebec).

Insulin was measured using a radioimmunoassay kit (Linco; St-Charles, MO), while adiponectin and leptin were measured using ELISA kits (Millipore, St-Charles, MO).

### 2.4. Histological Assessment

Liver samples obtained from each mouse were fixed in 10% formalin solution, embedded in paraffin, cut into sections, then mounted on glass slides, and stained with hematoxylin phloxine saffron (HPS). Liver steatosis was assessed according to the percentage of hepatic cells that exhibited macrovesicular fat droplets as follows: grade 0, absent, less than 5% of hepatocytes; grade 1, light, 5–33% of hepatocytes; grade 2, moderate, 33–66% of hepatocytes; grade 3, severe, >66% of hepatocytes affected [18].

### 2.5. Determination of Tissue Triglycerides (TG)

Tissue (100 mg) was powdered under liquid nitrogen and total lipids were extracted with 50 volumes of Folch reagent (2 : 1 chloroform-methanol) [19]. TG content was determined by using a commercial kit (Randox Laboratories Ltd., UK).

### 2.6. Western Blot Analysis

Immunoblotting was performed as previously described. Briefly, frozen muscle and liver tissues were homogenized in RIPA lysis buffer (25 mM Tris-HCl pH 7.4, 25 mM NaCl, 0.5 mM EDTA, 1% Triton-X-100, and 0.1% SDS) containing a protease inhibitor cocktail (Roche, Mannheim, Germany) as well as 1 mM phenylmethanesulfonyl fluoride and phosphatase inhibitors (1 mM sodium orthovanadate, 10 mM sodium pyrophosphate, and 10 mM sodium fluoride). The homogenate was centrifuged at 12,000 g at 4°C for 30 min. Aliquots of the supernatant were diluted to a concentration of 1.25 mg/mL total protein in reducing sample buffer (50 mM Tris-HCl pH 6.8, 1% SDS, 10% glycerol, 1% *β*-mercaptoethanol, and 0.01% bromophenol blue) and boiled for 5 min. Samples (20 *μ*L/well) were subjected to SDS-polyacrylamide gel electrophoresis (PAGE) on 10% polyacrylamide gels and transferred to nitrocellulose membranes (Millipore, Bedford, MA). Membranes were blocked for 2 h at room temperature with Tween-20 and 5% skimmed milk in TBS (20 mM Tris-HCl, pH 7.6, and 137 mM NaCl). Membranes were then incubated overnight at 4°C in blocking buffer with appropriate phospho-specific or pan-specific antibodies against AMPK, ACC, GLUT4, and acetyl-p53 (Lys 379) (each at 1 : 1000, Cell Signaling Technologies, Danvers, MA, USA) and PPAR*α* (1 : 200). Membranes were washed 5 times and incubated 1.5 h at room temperature in TBS plus Tween-20 with anti-rabbit HRP-conjugated secondary antibodies at 1 : 50000 to 1 : 100000 (Jackson Immunoresearch, Cedarlane Laboratories, Hornby, ON, Canada). Revelation was performed using the enhanced chemiluminescence method and blue-light-sensitive film (Amersham Biosciences, Buckinghamshire, England). Gel band intensities were evaluated by densitometric analysis using ImageJ densitometry software (Version 1.6, National Institutes of Health, Bethesda, MD, USA).

### 2.7. Statistical Analysis

The data were analyzed by SigmaStat 3.1 software (Jandel Scientific, San Rafael, CA) using one-way analysis of variance (ANOVA). Areas under the curve (AUC) were calculated by using PRISM software (GraphPad, San Diego, CA, USA). Nonparametric data was analyzed by the chi-square test. Statistical significance was set at *P* ≤ 0.05. Results are presented as the mean ± SEM for the indicated number of determinations or animals.

## 3. Results

### 3.1. *V. vitis-idaea* Significantly Improves HFD Induced Hyperglycemia in DIO Mice

As expected, control DIO animals were obese, hyperglycemic, hyperinsulinemic, and dyslipidemic (Tables 1 and 2). The observed leptin: adiponectin ratios and hepatic steatosis were also consistent with the establishment of an insulin-resistant state (Tables 2 and 3).

**Figure 1:**
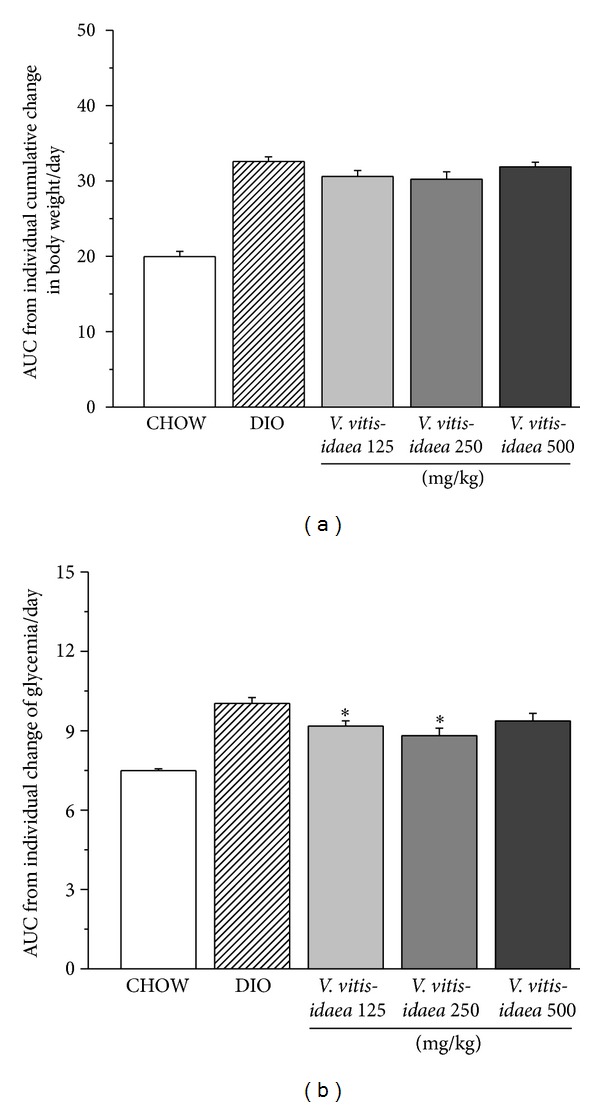
*V. vitis-idaea *treatment for 8 weeks reduces glycemia with little effect on body weight of DIO mice. (a) Cumulative change in body weight. (b) Nonfasting blood glucose concentration. These parameters were recorded three times per week. ∗ denotes significant difference as compared to control DIO (*P* < 0.05) as assessed by ANOVA test; *n* = 12 for each group.

*V. vitis-idaea* treatment had no effect on total body weight or retroperitoneal and epididymal fat weight (Figure 1(a) and Table 1). Similarly, caloric intake remained unchanged in the DIO control and treated groups as compared to the CHOW group (data not shown). Nevertheless,* V. vitis-idaea* treatment, given over the last eight-week period of experimental protocols at doses of 125 and 250 mg/kg, significantly decreased the area under the curve (AUC) of blood glucose levels by 9 and 12%, respectively, as compared to DIO controls (Figure 1(b); *P* < 0.05). On the other hand* V. vitis-idaea* at 500 mg/kg resulted in a weaker effect (7% reduction in glycemia) that failed to reach statistical significance. The antihyperglycemic effect was even more evident at the end of the treatments for the three doses where a significant drop was recorded (28%, 25%, and 17% decrease for the 125, 250, and 500 mg/kg doses, resp.; *P* < 0.05).

On the other hand, insulinemia was not significantly affected by the various treatments. A trend toward reduction (30%) compared to DIO controls was observed only in the 500 mg/kg* V. vitis-idaea*-treated group (Table 2).

### 3.2. *V. vitis-idaea* Treatment Attenuates Hepatic Steatosis and Hyperlipidemia in DIO Mice

Hepatic steatosis was evaluated by histological scoring of liver tissue sections as previously validated [18]. As expected, 82% of the animals in the DIO control group exhibited severe (grade 3) steatosis, while 18% had mild to moderate (grade 1 or 2) steatosis, as compared with 100% healthy livers (grade 0) observed in nonobese CHOW-fed congeners.* V. vitis-idaea* treatment decreased the proportion of DIO animals exhibiting grade 3 steatosis to only 50–66%. Interestingly, animals with healthy livers (grade 0 steatosis), which were nonexistent in the control DIO group, were present in proportions ranging between 8 and 33% in* V. vitis-idaea*-treated DIO animals. On the other hand, 20–32% of* V. vitis-idaea* group exhibited either grade 1 or grade 2 steatosis (Table 3; *P* < 0.05), confirming overall improvement in this parameter. The group receiving the 250 mg/kg/d dose showed the best reduction in steatotic histological profile.

**Figure 2:**
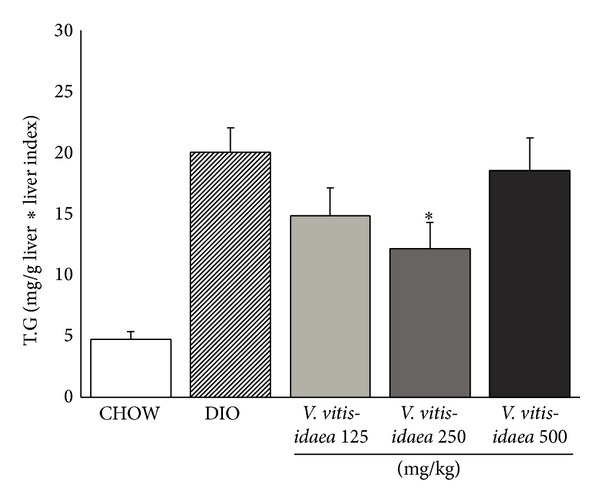
*V. vitis-idaea* treatment decreases triglyceride content (TG) in the liver of DIO mice. The colorimetric dosage of TG levels (*n* = 12) was determined using a commercial kit. Data are presented as the mean ± SEM and representative of 12 mice per experimental group. ∗ indicates a *P* value < 0.05 significantly different from CHOW group.

Consistent with these results,* V. vitis-idaea* reduced liver triglyceride levels. Both the 125 and the 250 mg/kg/d groups demonstrated a statistically significant reduction (39%) (Figure 2; *P* < 0.05). Moreover, the same 250 mg/kg/d dose was able to significantly reduce total plasma cholesterol and plasma LDL by 12% and 18%, respectively (Table 2; *P* < 0.05).

### 3.3. *V. vitis-idaea *Improves Insulin Signaling, Tends to Activate AMPK and SIRT1, and Increases Total Protein Content of GLUT4 in Skeletal Muscle of DIO Mice

**Figure 3:**
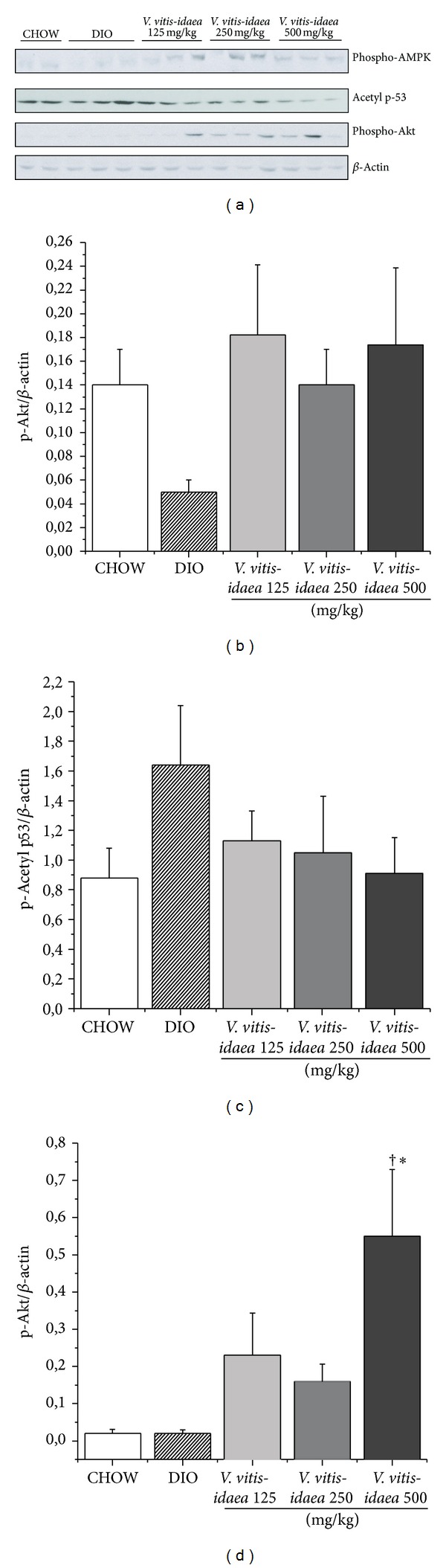
*V. vitis-idaea* activates Akt and AMPK pathways and tends to decrease acetyl p53 content in soleus muscle of DIO mice. Samples of soleus muscles were obtained from CHOW, DIO control, and DIO mice fed with* V. vitis-idaea* (125, 250, and 500 mg/Kg) and analyzed by immunoblotting. (a) Representative blots for each group are shown for samples probed with p-Akt, p-AMPK, acetyl p53, and *β*-actin as loading control. Data are expressed as mean ± SEM from 12 animals in each experimental group for (b) p-Akt (Ser 473)/*β*-actin, (c) p-AMPK*α* (Thr 172)/*β*-actin, and (d) Acetyl p53 (Lys 379)/*β*-actin. ∗ indicates a *P* value ≤ 0.05 significantly different from CHOW group, and † indicates a *P* value ≤ 0.05 significantly different from DIO control group.

**Figure 4:**
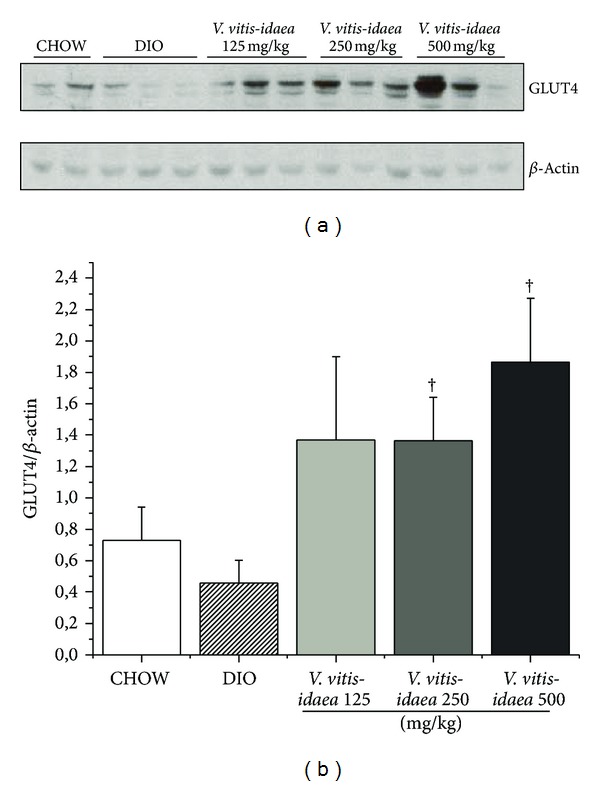
*V. vitis-idaea* increases GLUT4 protein content in soleus muscle of DIO mice. Samples of soleus muscles were obtained from CHOW, DIO control, and DIO mice fed with* V. vitis-idaea* (125, 250, and 500 mg/Kg) and analysed by immunoblotting with antibodies specific to GLUT4. (a) Representative blots for each group are shown. (b) Data are expressed as GLUT4/*β*-actinand are presented as mean ± SEM from 12 animals per experimental group. † indicates a *P* value < 0.05 which is significantly different from DIO control group.

When compared to DIO control group, western blots for muscle of DIO mice fed with* V. vitis-idaea* revealed that the low, medium, and high doses showed a strong tendency to increase AMPK phosphorylation (Figures 3(a) and 3(b); *P* = 0.057). On the other hand, the high dose of the plant extract showed a tendency to decrease the content of acetylated p53 (Lys 379) (Figures 3(a) and 3(c); *P* = 0.11) and significantly increased the phosphorylation of Akt (Serine 473) (Figures 3(a) and 3(d); *P* < 0.05). Moreover, GLUT4 protein levels were significantly increased by 1.4- to 2-fold in DIO mice fed with the medium and high dose of* V. vitis-idaea* (Figures 4(a) and 4(b); *P* < 0.05).

### 3.4. *V. vitis-idaea* Activates Insulin and AMPK Signaling Pathways in the Liver of DIO Mice

**Figure 5:**
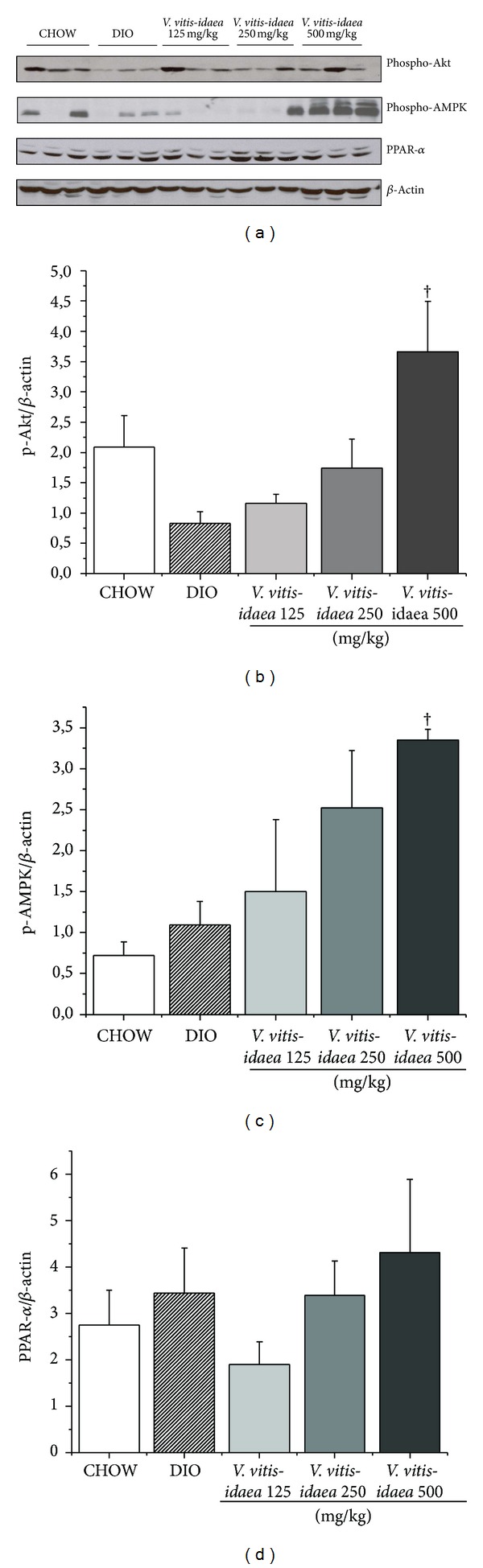
*V. vitis-idaea* treatment activates Akt and AMPK pathway but does not increase PPAR-*α* content in the liver of DIO mice. Samples of liver tissue from CHOW, control DIO, and DIO mice fed with* V. vitis-idaea* (125, 250, and 500 mg/Kg) were homogenized and analysed by immunoblotting. (a) Representative immunoblots of samples probed with p-AMPK, p-Akt, PPAR*α*, and *β*-actin as loading control are shown. Data are expressed as mean ± SEM from 12 animals per experimental group for (b) p-AMPK/*β*-actin, (c) p-Akt/*β*-actin,** and** (d) PPAR*α*/*β*-actin andlevels were quantified using densitometry. † indicates a *P* value < 0.05 significantly different from DIO control group.

A dose dependent activation of both insulin and AMPK pathways was recorded as increases in the phosphorylation of Akt and AMPK, respectively, in liver of DIO mice fed with* V. vitis-idaea* extract, but only the high dose group reached statistical significance (Figures 5(a), 5(b), and 5(c); *P* < 0.05). This was not associated with an increase in hepatic content of PPAR-*α*, a key transcription factor controlling hepatic fatty acid oxidation (N.S., Figures 5(a) and 5(d)), nor with any changes in acetylated p53 (not illustrated).

## 4. Discussion

Due to the adoption of more sedentary lifestyles and a gradual transition from traditional to western-type diets by Aboriginal populations in Canada, there has been an upsurge in the prevalence of obesity and diabetes, two of the most serious threats to global public health [8, 20, 21]. Health problems of Aboriginal populations are confounded by the cultural disconnection of modern drug-based therapies, leading to difficulties with compliance and enhanced diabetes complications [16]. In an effort to develop culturally adapted treatment options for Cree diabetics, our research team has been examining several plants of the Canadian boreal forest stemming from Cree traditional medicine.

Lingonberry (*V. vitis-idaea*) was identified through an ethnobotanical study conducted in collaboration with the Cree communities of Eeyou Istchee (northern Quebec, Canada) [14] and exhibited a potent and promising antidiabetic activity in cell-based* in vitro* bioassays [15, 17]. The current studies sought to evaluate this antidiabetic potential* in vivo*. For this purpose, models of diet-induced obesity are widely used as nongenetic models for type 2 diabetes research to mimic the more common form of human obesity-induced diabetes (coined by some as diabesity). For instance, male DIO C57BL/6J mice fed a high-fat diet share many obesity phenotypes with humans, such as abdominal adiposity, hyperinsulinemia, insulin resistance, and fatty liver [22]. Therefore, this mouse model was chosen to further investigate the antidiabetic and antiobesity effect of* V. vitis-idaea *berries.

Mice fed the HFD regimen for a period of 8 weeks establish an obesity-induced prediabetic state, as confirmed herein. It is at that time-point that low, medium, or high doses of* V. vitis-idaea *(125, 250, 500 mg/kg) were added to HFD and administered over the subsequent 8-week treatment period. The results of this study clearly show that* V. vitis-idaea* possesses an antihyperglycemic effect despite the continued intake of HFD. Indeed, the attenuation of hyperglycemia and dyslipidemia provides evidence of improved insulin resistance in* V. vitis-idaea*-treated DIO groups. These effects were more pronounced as the dose of the plant extract rose from 125 to 250 mg/kg and yet declined thereafter. Such a “bell-shaped” dose-response relationship is not unusual for pharmacological agents, particularly medicinal plants [23].

*V. vitis-idaea *treatment did not affect caloric intake and showed only a mild tendency to reduce body weight at the highest extract dose. It is noteworthy that* V. vitis-idaea* was able to significantly reduce glycemia despite unchanged cumulative food intake and body weight. Importantly,* V. vitis-idaea*-treated animals, even at the highest dose of 500 mg/kg/d, showed no signs of toxicity, as evidenced by a lack of behavioral changes. In addition, parameters of hematological, liver, and kidney function were similar to DIO controls. The safety of this plant is further supported by the GRAS (generally regarded as safe) status of the edible berries, being widely consumed by the CEI and other populations. Hence, it is highly unlikely that observed improvements in systemic glucose homeostasis are related to nonspecific toxic actions of the plant extracts.

To further understand the underlying mechanisms of the metabolic effect of* V. vitis-idaea* and to begin identifying molecular targets, we monitored the change in the expression and/or activity of key proteins involved in glucose and lipid metabolism in skeletal muscle and liver, the principal organs responsible for regulation of glucose and lipid homeostasis. Our results demonstrate that* V. vitis-idaea* enhances both insulin-dependent and -independent pathways in HFD-fed DIO mice.

In skeletal muscle, phosphorylation of both Akt and, to a lesser extent, AMPK was observed, indicating an elevation in the activity of both kinases. The enhancement of both insulin-dependent and -independent kinases was associated with increased muscle expression of the effector protein GLUT4, thereby providing a feasible explanation for the antihyperglycemic effects of* V. vitis-idaea*. Indeed, GLUT4-dependent muscle glucose uptake contributes significantly to peripheral glucose disposition and hence to reduced glycemia [24].

We also evaluated SIRT1-mediated deacetylation of p53, since the latter has been used as an indicator for SIRT1 activity [25]. Consistent with the previously reported decrease in SIRT1 activity induced by high caloric diets [6], levels of muscle acetyl p53 tended to increase in control DIO mice, whereas* V. vitis-idaea* treatment had the opposite effect. Enhanced SIRT1 activity may thus participate in the beneficial action of the plant extract on muscle energy metabolism, although further studies are needed to confirm the tendencies observed herein.

We next assessed components involved in liver intermediate metabolism. Indeed, in obese individuals, uninhibited lipolysis in insulin-resistant adipose tissue leads to increased free fatty acid delivery to the liver [22]. The resulting abnormal fat accumulation in the liver (hepatic steatosis) induces hepatic insulin resistance, impairing insulin-mediated inhibition of hepatic glucose output and increasing the risk for type 2 diabetes [26]. In the present study, the most effective antihyperglycemic dose of* V. vitis-idaea* significantly lowered liver triglyceride content and the histological grade of hepatic steatosis, which is also consistent with reduced serum levels of LDL and total cholesterol. It is thus likely that* V. vitis-idaea *treatment improved liver insulin sensitivity. This was corroborated by the enhanced liver activation of insulin-dependent Akt recorded herein.

*V. vitis-idaea* also enhanced liver AMPK activation. However, neither the expression of the PPAR-*α* protein nor the acetylation of p53 (both involved in the control of hepatic lipid oxidation) was affected by the plant extract. Hence,* V. vitis-idaea* treatment may improve hepatic lipid homeostasis through the activation of AMPK and insulin signaling pathways but does not appear to involve PPAR-*α* or SIRT-1 related components.

In summary, dietary intake of* V. vitis-idaea* berry extract helps normalize blood glucose in HFD-fed DIO mice without significantly affecting food intake or body weight. Its mechanism of action involves enhanced expression of GLUT4 in skeletal muscle and diminished hepatic steatosis, both through insulin-dependent and -independent pathways. Together with our previous* in vitro* study, these collective findings confirm the significant antidiabetic potential of* V. vitis-idaea* berries. Our work thus supports the use of this medicinal plant in the context of culturally sensitive dietary and therapeutic interventions for type 2 diabetes in Aboriginal populations of Quebec.

## Figures and Tables

**Table 1:** Weight of body and various organs of DIO mice treated with *V. vitis-idaea*.

	CHOW	DIO control	*V. vitis-idaea *		
			125 mg/kg	250 mg/kg	500 mg/kg
Body weight (g)	36.5 ± 1.0	47.6 ± 0.1*	45.1 ± 1.3*	43.9 ± 1.6*	47.3 ± 1.0*
Liver weight (g)	1.6 ± 0.1	2.4 ± 0.1*	2.2 ± 0.2*	2.0 ± 0.2^†^	2.4 ± 0.2*
Retroperitoneal fat (g)	0.8 ± 0.0	1.7 ± 0.1*	1.8 ± 0.1*	1.4 ± 0.1*	1.8 ± 0.1*
Epididymal fat (g)	1.9 ± 0.1	1.5 ± 0.1	1.8 ± 0.1*	1.5 ± 0.1	1.4 ± 0.1
Brown fat (g)	0.3 ± 0.0	0.5 ± 0.0*	0.5 ± 0.1*	0.5 ± 0.0*	0.5 ± 0.0*

All values are mean ± SEM (*n* = 12). *denotes significant difference as compared to control CHOW, and ^†^indicates significant difference from DIO control group (*P* ≤ 0.05) as assessed by ANOVA test.

**Table 2:** Blood parameters at the end of the treatment.

	CHOW	Control DIO	*V.vitis-idaea *		
			125 mg/kg	250 mg/kg	500 mg/kg
Triglycerides (mmol/L)	0.83 ± 0.08	0.74 ± 0.03	0.65 ± 0.04	0.67 ± 0.06	0.72 ± 0.04
Total cholesterol (mmol/L)	1.76 ± 0.15	3.34 ± 0.13*	3.09 ± 0.19*	2.96 ± 0.16^∗†^	3.46 ± 0.15*
HDL (mmol/L)	0.69 ± 0.1	1.28 ± 0.04*	1.30 ± 0.07*	1.23 ± 0.07*	1.34 ± 0.03*
LDL (mmol/L)	0.69 ± 0.04	1.7 ± 0.1*	1.72 ± 0.09*	1.49 ± 0.11^∗†^	1.77 ± 0.11*

Insulin (ng/mL)	4.48 ± 0.81	41.96 ± 18.88	43.71 ± 22.79	40.45 ± 15.45	29.11 ± 8.78
Leptin (ng/mL)	25.97 ± 1.52	34.09 ± 1.60*	34.20 ± 1.86*	35.65 ± 2.22*	36.94 ± 0.92*
Adiponectin (µg/mL)	17.29 ± 0.54	13.44 ± 0.96*	14.57 ± 0.82*	15.35 ± 1.60*	13.87 ± 1.13*
Leptin/adiponectin	1.50 ± 0.07	2.64 ± 0.20*	2.37 ± 0.12*	2.50 ± 0.25*	2.90 ± 0.32*

ALT (U/L )	26.18 ± 2.71	37.09 ± 3.47*	34.66 ± 4.47*	32.18 ± 4.90*	45.66 ± 6.50*
AST (U/L)	123.64 ± 23.50	109.45 ± 8.70*	98.50 ± 12.58*	102.54 ± 8.22*	116.16 ± 11.17
Creatinine (U/L)	51.40 ± 4.40	53.09 ± 5.11	59.83 ± 11.38^†^	55.81 ± 6.59	64.50 ± 11.35^†^
Alc. phosphatase (U/L)	35.45 ± 2.58	64.90 ± 20.00*	39.66 ± 3.06^†^	39.63 ± 3.63^†^	47.83 ± 3.82^†^

All values are mean ± SEM (*n* = 12). *indicates a *P* value < 0.05 which is significantly different from CHOW group, and ^†^indicates a *P* value < 0.05 which is significantly different from DIO control group (*P* ≤ 0.05) as assessed by ANOVA test.

**Table 3:** Histological scores of liver steatosis from DIO mice treated with *V. vitis-idaea*.

Groups	*n*	Grades of steatosis			
		0	1	2	3
CHOW	11	11	0	0	0
Control DIO	11	0	1	1	9
*V. vitis-idaea* 125 mg/kg	12	2	2	2	6
*V. vitis-idaea* 250 mg/kg	12	4	0	1	7
*V. vitis-idaea* 500 mg/kg	12	1	2	1	8

The scoring is based on the percentage of hepatocytes containing macrovesicular steatosis, grade0: 0–5%, grade 1: 5–33%, grade 2: 33–66%, and grade 3: more than 66% (21) (chi-square; *P* < 0.05).

## References

[1] Marette A, Burdett E, Douen A, Vranic M, Klip A (1992). Insulin induces the translocation of GLUT4 from a unique intracellular organelle to transverse tubules in rat skeletal muscle. *Diabetes*.

[2] Pelletier A, Joly E, Prentki M, Coderre L (2005). Adenosine 5′-monophosphate-activated protein kinase and p38 mitogen-activated protein kinase participate in the stimulation of glucose uptake by dinitrophenol in adult cardiomyocytes. *Endocrinology*.

[3] Fogarty S, Hardie DG (2010). Development of protein kinase activators: AMPK as a target in metabolic disorders and cancer. *Biochimica et Biophysica Acta*.

[4] Zhou G, Sebhat IK, Zhang BB (2009). AMPK activators—potential therapeutics for metabolic and other diseases. *Acta Physiologica*.

[5] Viollet B, Andreelli F, Jorgensen SB (2003). Physiological role of AMP-activated protein kinase (AMPK): insights from knockout mouse models. *Biochemical Society Transactions*.

[6] Boily G, Seifert EL, Bevilacqua L (2008). SirT1 regulates energy metabolism and response to caloric restriction in mice. *PLoS ONE*.

[7] Yamazaki Y, Usui I, Kanatani Y (2009). Treatment with SRT1720, a SIRT1 activator, ameliorates fatty liver with reduced expression of lipogenic enzymes in MSG mice. *American Journal of Physiology: Endocrinology and Metabolism*.

[8] Hegele RA (2001). Genes, environment and diabetes in Canadian aboriginal communities. *Advances in Experimental Medicine and Biology*.

[9] Hjalmasson I, Ortiz R, Janick JJ (2001). Lingonberry: botany and horticulture. *Horticultural Reviews*.

[10] Wang SY, Feng R, Bowman L, Penhallegon R, Ding M, Lu Y (2005). Antioxidant activity in lingonberries *(Vaccinium vitis-idea L.)* and its inhibitory effect on activator protein-1, nuclear factor-kappaB, and mitogen-activated protein kinases activation. *Journal of Agricultural and Food Chemistry*.

[11] Heller CA (1953). *Edible and Poisonous Plants of Alaska*.

[12] Leighton AL (1985). *Wild Plant Use by the Woods Cree (Nihithawak) of East-Central Saskatchewan*.

[13] Fraser MH, Cuerrier A, Haddad PS, Arnason JT, Owen PL, Johns T (2007). Medicinal plants of Cree communities (Quebec, Canada): antioxidant activity of plants used to treat type 2 diabetes symptoms. *Canadian Journal of Physiology and Pharmacology*.

[14] Leduc C, Coonishish J, Haddad P, Cuerrier A (2006). Plants used by the Cree Nation of Eeyou Istchee (Quebec, Canada) for the treatment of diabetes: a novel approach in quantitative ethnobotany. *Journal of Ethnopharmacology*.

[15] Harbilas D, Martineau LC, Harris CS (2009). Evaluation of the antidiabetic potential of selected medicinal plant extracts from the Canadian boreal forest used to treat symptoms of diabetes: part II. *Canadian Journal of Physiology and Pharmacology*.

[16] Bullen JW, Ziotopoulou M, Ungsunan L (2004). Short-term resistance to diet-induced obesity in A/J mice is not associated with regulation of hypothalamic neuropeptides. *American Journal of Physiology: Endocrinology and Metabolism*.

[17] Eid HM, Martineau LC, Saleem A (2010). Stimulation of AMP-activated protein kinase and enhancement of basal glucose uptake in muscle cells by quercetin and quercetin glycosides, active principles of the antidiabetic medicinal plant *Vaccinium vitis-idaea*. *Molecular Nutrition and Food Research*.

[18] Brunt EM, Janney CG, di Bisceglie AM, Neuschwander-Tetri BA, Bacon BR (1999). Nonalcoholic steatohepatitis: a proposal for grading and staging the histological lesions. *The American Journal of Gastroenterology*.

[19] Folch J, Lees M, Sloane Stanley GH (1957). A simple method for the isolation and purification of total lipides from animal tissues. *The Journal of Biological Chemistry*.

[20] Young TK, Reading J, Elias B, O’Neil JD (2000). Type 2 diabetes mellitus in Canada’s First Nations: status of an epidemic in progress. *Canadian Medical Association Journal*.

[21] Katzmarzyk PT (2008). Obesity and physical activity among Aboriginal Canadians. *Obesity*.

[22] Ha SK, Chae C (2010). Inducible nitric oxide distribution in the fatty liver of a mouse with high fat diet-induced obesity. *Experimental Animals*.

[23] Ni W, Zhang X, Wang B (2010). Antitumor activities and immunomodulatory effects of ginseng neutral polysaccharides in combination with 5-fluorouracil. *Journal of Medicinal Food*.

[24] Eid HM, Ouchfoun M, Brault A (2011). W9, a medicinal plant from the pharmacopeia of the Eastern James Bay Cree, exihibits anti-diabetic activities in two mouse model of diabetes. *Planta Medica*.

[25] Kang H, Jung JW, Kim MK, Chung JH (2009). CK2 is the regulator of SIRT1 substrate-binding affinity, deacetylase activity and cellular response to DNA-damage. *PLoS ONE*.

[26] Qureshi K, Abrams GA (2007). Metabolic liver disease of obesity and role of adipose tissue in the pathogenesis of nonalcoholic fatty liver disease. *World Journal of Gastroenterology*.

